# Adipose stem cell crosstalk with chemo-residual breast cancer cells: implications for tumor recurrence

**DOI:** 10.1007/s10549-018-05103-w

**Published:** 2018-12-29

**Authors:** Matthew A. Lyes, Sturgis Payne, Paul Ferrell, Salvatore V. Pizzo, Scott T. Hollenbeck, Robin E. Bachelder

**Affiliations:** 1 Department of Pathology, Duke University Medical Center, Durham, NC USA; 2 Division of Plastic and Reconstructive Surgery, Department of Surgery, Duke University Hospital, Durham, NC USA; 3 308 Research Drive, LSRC B217, Durham, NC 27710 USA

**Keywords:** Triple-negative breast cancer (TNBC), Adipose-derived stem cells (ASCs), Migration, Proliferation, Fibroblast growth factor 2 (FGF2), Recurrence

## Abstract

**Purpose:**

Most triple-negative breast cancer (TNBC) patients exhibit an incomplete response to neoadjuvant chemotherapy, resulting in chemo-residual tumor cells that drive tumor recurrence and patient mortality. Accordingly, strategies for eliminating chemo-residual tumor cells are urgently needed. Although stromal cells contribute to tumor cell invasion, to date, their ability to influence chemo-residual tumor cell behavior has not been examined. Our study is the first to investigate cross-talk between adipose-derived stem cells (ASCs) and chemo-residual TNBC cells. We examine if ASCs promote chemo-residual tumor cell proliferation, having implications for tumor recurrence.

**Methods:**

ASC migration toward chemo-residual TNBC cells was tested in a transwell migration assay. Importance of the SDF-1α/CXCR4 axis was determined using neutralizing antibodies and a small molecule inhibitor. The ability of ASCs to drive tumor cell proliferation was analyzed by culturing tumor cells ± ASC conditioned media (CM) and determining cell counts. Downstream signaling pathways activated in chemo-residual tumor cells following their exposure to ASC CM were studied by immunoblotting. Importance of FGF2 in promoting proliferation was assessed using an FGF2-neutralizing antibody.

**Results:**

ASCs migrated toward chemo-residual TNBC cells in a CXCR4/SDF-1α-dependent manner. Moreover, ASC CM increased chemo-residual tumor cell proliferation and activity of extracellular signal-regulated kinase (ERK). An FGF2-neutralizing antibody inhibited ASC-induced chemo-residual tumor cell proliferation.

**Conclusions:**

ASCs migrate toward chemo-residual TNBC cells via SDF-1α/CXCR4 signaling, and drive chemo-residual tumor cell proliferation in a paracrine manner by secreting FGF2 and activating ERK. This paracrine signaling can potentially be targeted to prevent tumor recurrence.

## Introduction

Approximately 170,000 cases of triple-negative breast cancer (TNBC) are diagnosed in the US annually [1]. These tumors do not express estrogen receptor, progesterone receptor, or HER2, making them unresponsive to currently available targeted therapies [2]. Although many of these tumors initially respond to chemotherapy, over 60% of cases exhibit an incomplete pathologic response, yielding high rates of 5-year recurrence [3] and patient mortality [4].

We previously showed that TNBC is composed of heterogeneous tumor cell subpopulations, only some of which are responsive to chemotherapy [5, 6]. Following chemotherapy treatment, chemo-sensitive cells die off, initially shrinking the tumor. However, a residual population of chemotherapy-resistant cells remains and can be detected post-treatment. These chemo-residual tumor cells are usually dormant, and can remain in that state for years prior to resuming proliferation and driving tumor recurrence. Recurrent tumors are usually responsible for patient mortality and they are resistant to current therapies. Accordingly, there exists a need to identify signaling axes that can be targeted to prevent proliferation of chemo-residual tumor cells.

Adipose-derived stem cells (ASCs) are found in association with tumors in vivo and are recruited from abundant breast adipose tissue to the tumor microenvironment via poorly understood signaling pathways [7]. Previous work demonstrates that ASCs drive breast tumor cell growth [8, 9] and increase their metastatic potential in vivo [10]. However, to date, it remains unclear if ASCs migrate toward and support the growth of chemo-residual tumor cells, thus potentially contributing to tumor recurrence.

It is known that ASC chemotaxis is dependent on the binding of stromal derived factor 1α (SDF-1α), which is secreted by target tissues, to the C-X-C chemokine receptor type 4 (CXCR4) on the ASC cell surface [11]. In the current work, we address the hypothesis that following cessation of chemotherapy treatment, chemo-residual TNBC cells recruit ASCs to their vicinity by secreting SDF-1α. Once within the tumor microenvironment, we show that these ASCs induce the proliferation of chemo-resistant TNBC cells. Our results suggest that ASCs may contribute to tumor progression in TNBC patients exhibiting an incomplete pathologic response to neoadjuvant chemotherapy treatment.

## Results

### ASC migratory potential toward chemo-residual TNBC cells

**Fig. 1:**
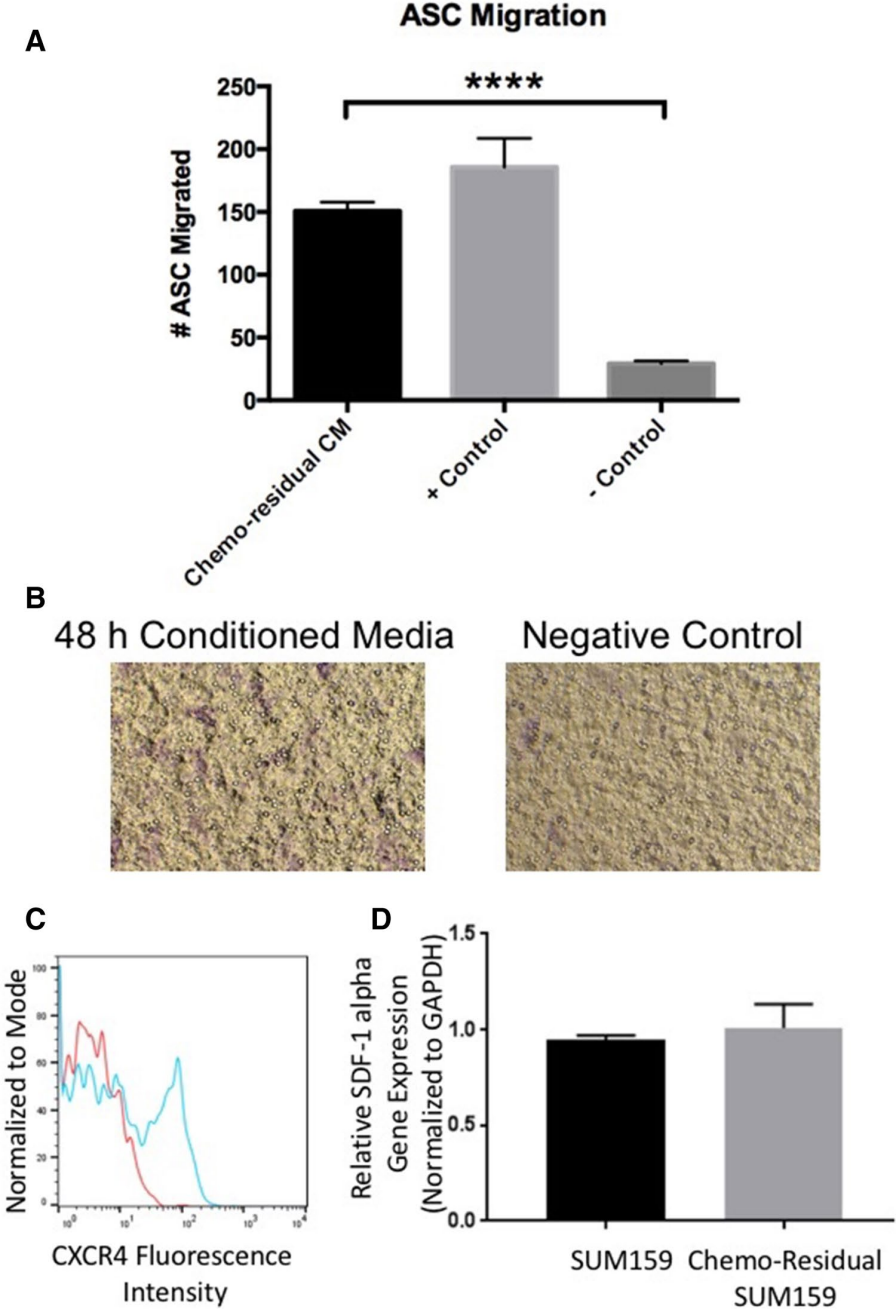
Adipose stem cells (ASCs) Migrate toward Conditioned Media (CM) from Chemo-Residual TNBC Cells. **A** CM was prepared by growing chemo-residual SUM159 TNBC cells in serum-free media for 48 h. The ability of primary human ASCs (Zenbio) to migrate toward this CM (Chemo-residual CM), FBS (+ Control) or serum-free media (− Control) was evaluated in a 15 h transwell assay. Total number of migrated cells from 5 representative fields (100 × magnification) was determined for each well, and mean cell number from triplicate wells (± SD) was calculated. Significance was determined by two tailed *t* test (****p < 0.0001). Similar results were obtained in at least three independent trials. **B** Representative fields (100 ×) showing migration of ASCs towards chemo-residual TNBC CM in a 15 h transwell assay. **C** CXCR4 expression was assessed by incubating ASCs with CXCR4 antibody (blue line) or control IgG (red line), followed by FITC-conjugated secondary antibody. CXCR4 cell surface expression was determined by flow cytometry. **D** RNA was isolated from untreated and chemo-residual SUM159 cells and subjected to SDF-1 alpha and GAPDH real-time PCR. SDF-1 alpha gene expression relative to GAPDH is presented for both cell lines

In order to develop an understanding of how adipose stroma impacts chemo-residual TNBC cells, we first sought to show that ASCs migrate along a chemotactic axis to localize to chemo-residual TNBC cells. We obtained primary human ASCs from Zenbio. These cells were shown by Zenbio to express stem cell surface markers, as well as the ability to differentiate into adipocytes, chondrocytes, and osteocytes. Multilineage differentiation potential of these cells was confirmed in our laboratory (data not shown). Chemo-residual TNBC cells were generated using our short-term chemotherapy treatment model [5]. In this model, TNBC cells were exposed to a clinically-relevant chemotherapy regimen (docetaxel) for 2 days, after which drug was removed from the culture media. By day 7, we observed a subpopulation of growth-arrested tumor cells surviving chemotherapy. Approximately 2 weeks after chemotherapy removal, this chemo-residual tumor cell subpopulation resumed growth, establishing multi-drug resistant colonies. For the current study, conditioned media (CM) from these chemotherapy-resistant SUM159 TNBC cells was collected and placed into the bottom chamber of a transwell with ASCs seeded in the top chamber. Following a 15 h incubation, ASCs migrated toward chemo-residual TNBC conditioned media as demonstrated in Fig. 1A. We observed a four-fold increase in migration of ASCs exposed to chemo-residual TNBC CM relative to those exposed to serum free control media.

**Fig. 2:**
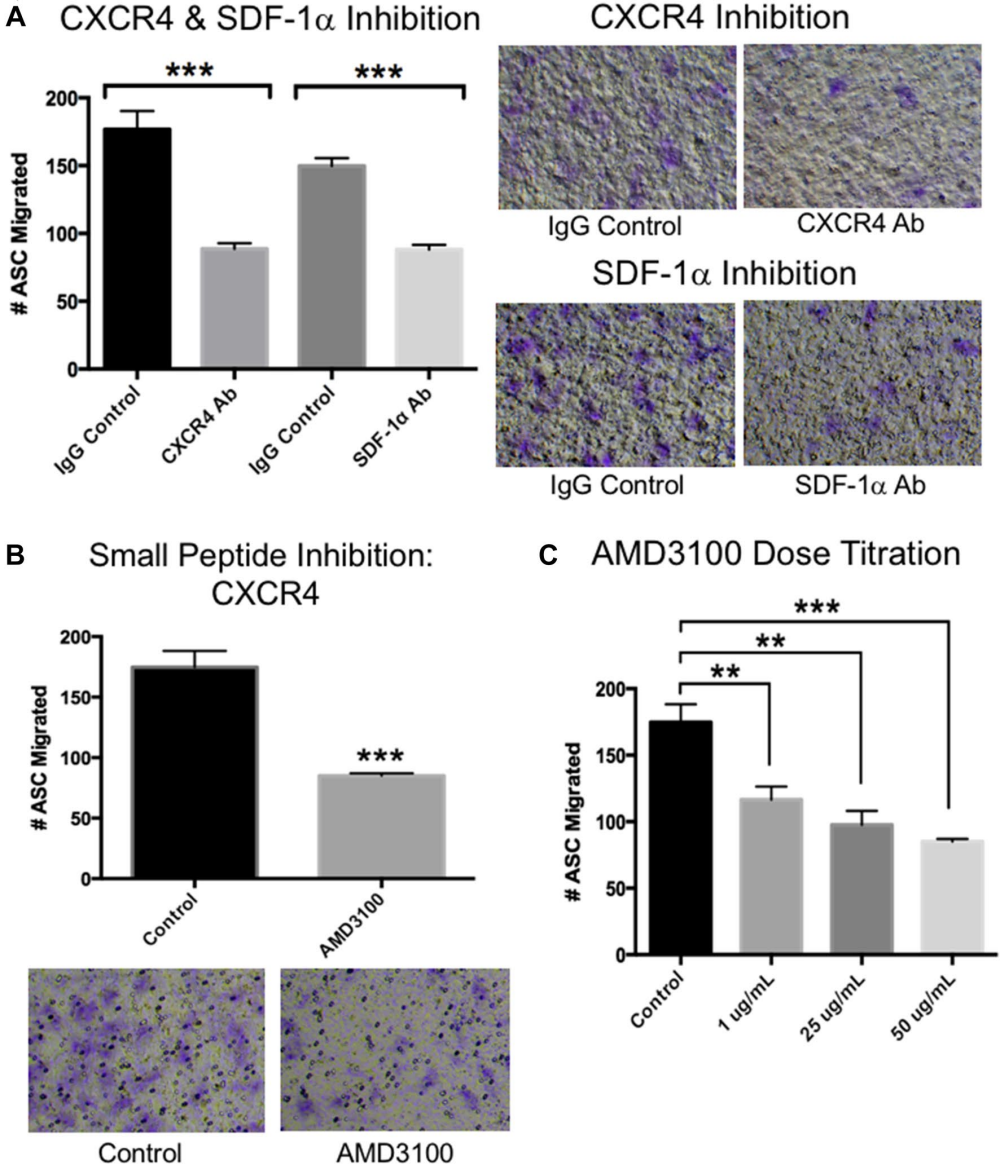
CXCR4 and SDF-1α Neutralizing Antibodies Block ASC Migration toward chemo-residual TNBC cell Conditioned Media. **A** Human ASC migration toward chemo-residual TNBC cell CM (prepared as described in Fig. 1) was measured in the presence of a CXCR4-neutralizing Ab (EMDMillipore, 10 µg/mL), an SDF1α -neutralizing antibody (R&D Systems, 10 µg/mL), or control IgG (R&D Systems, 10 µg/mL). Chemotaxis was measured as in Fig. 1. The right panels show representative fields of migrated ASCs in the presence of the indicated antibodies. Significance was measured with a two tailed *t* test (***p < 0.0005). **B** The ability of a CXCR4 small molecule inhibitor (AMD3100; EMD Millipore, 50 µg/mL) to block ASC migration toward chemo-residual TNBC cell CM was tested in a 15 h transwell assay. The bottom panels are representative fields of migrated ASCs ± AMD3100. Significance was measured with a two tailed *t* test (***p < 0.0005). **C** The ability of ASCs to migrate toward chemo-residual TNBC cell CM was tested in a 15 h transwell assay in the presence of the indicated concentrations of CXCR4 small molecule inhibitor (AMD3100). Significance was measured using a two tailed *t* test (**p < 0.005; ***p < 0.0005)

Previous studies indicate that ASCs migrate along the SDF1α-CXCR4 chemotactic axis [12]. First, we confirmed CXCR4 expression in our ASCs by flow cytometry (Fig. 1C). We also showed by real-time PCR that TNBC cells express SDF1α (Fig. 1D). To determine if this axis drives the migration of ASCs toward TNBC chemo-residual cells, we performed transwell chemotaxis assays that included either CXCR4 or SDF-1α neutralizing antibodies. As shown in Fig. 2, both antibodies reduced ASC migration toward chemo-residual TNBC (SUM159) conditioned media. We also showed that a CXCR4 small molecule inhibitor (AMD3100; EMD Milipore) reduced ASC migration toward chemo-residual cells by two-fold (Fig. 2B). The inhibitory effect of this peptide was greater with increasing inhibitor concentration (Fig. 2C). Notably, these antibodies and peptides only partially reduced the migratory effect of tumor cell conditioned media on ASC migration, suggesting that additional signaling axes participate in this biology.

### ASCs promote chemo-residual TNBC cell proliferation

**Fig. 3:**
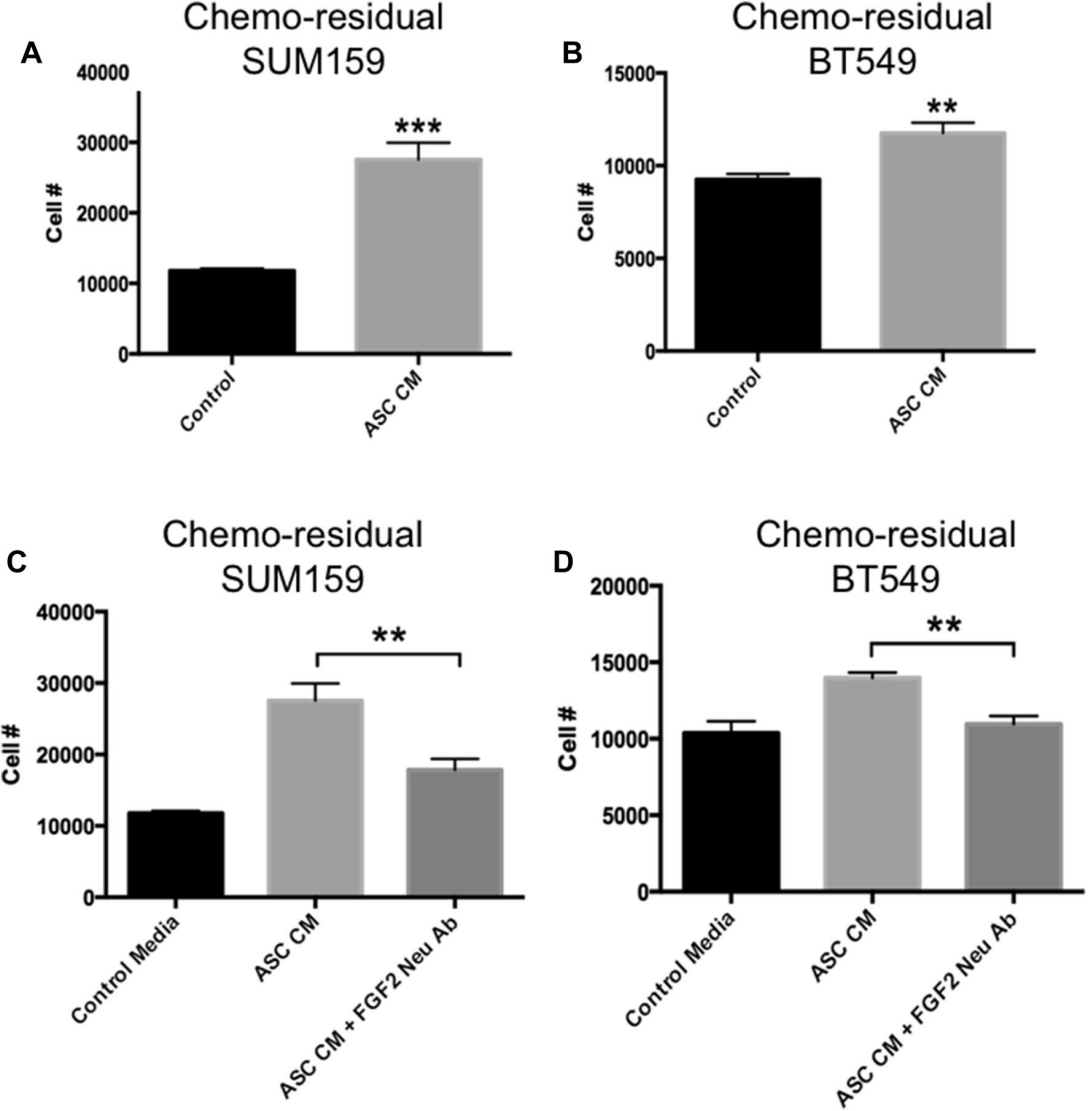
ASC CM Increases Proliferation of Chemo-residual TNBC Cells in an FGF2-dependent manner. **A** and **B** ASC CM was prepared from human ASCs (ZenBio) grown in reduced serum media for 72 h. This ASC CM (ASC CM), or control reduced serum media (Control) was added to chemo-residual SUM159 (**A**) or chemo-residual BT549 (**B**) cells. The number of chemo-residual TNBC cells was determined after 24 h by trypan blue staining. Results are reported as mean cell number (± SEM) from triplicate wells. Similar results were obtained in 3 trials. Significance was measured using a two tailed *t* test, ***p < 0.0005. (**C** and **D**) Chemo-residual SUM159 cells (**C**) or chemo-residual BT549 cells (**D**) were incubated with control media, ASC CM + control IgG, or ASC CM + FGF2-neutralizing antibody (EMD Millipore, 10 µg/mL) for 24 h. Cell numbers were determined as in A. Significance was measured with a two tailed *t* test (**p < 0.005). This effect was independently observed in three trials. Of note, incubation of chemo-naïve SUM159 cells with ASC CM did not induce cell proliferation (data not shown)

Having shown that ASCs can migrate toward chemo-residual TNBC cells, we next sought to determine if these ASCs influence the proliferation of chemo-residual TNBC cells, which exhibit reduced proliferation relative to untreated TNBC cells [6]. ASCs secrete growth factors that can act in a paracrine fashion on neighboring cells. Accordingly, we collected conditioned media from ASCs (ASC CM), and tested its effects on chemo-residual tumor cell growth. ASC CM increased chemo-residual TNBC cell number significantly after 24 h, as determined by trypan blue staining (Fig. 3A, B). Chemo-residual SUM159 cells exposed to ASC CM demonstrated a 2.3-fold increase in cell number relative to control (Fig. 3A), while chemo-residual BT549 cells demonstrated a 1.3-fold increase (Fig. 3B). Notably, we did not observe an ability of ASC CM to drive proliferation of chemo-naïve TNBC cells (data not shown).

Previous studies indicate that TNBC cells are dependent on fibroblast growth factor 2 (FGF2) for their growth and survival, which has led to the clinical use of FGFR tyrosine kinase inhibitors to slow primary tumor growth and progression [13, 14]. Based on the knowledge that ASCs secrete FGF2 [9], we next sought to determine if ASCs drive chemo-residual TNBC cell proliferation in an FGF2-dependent manner. ASC CM was added to chemo-residual TNBC cells in the presence of an FGF2-neutralizing antibody (or control IgG) for 24 h. Cell number was determined by trypan blue staining. FGF2 neutralizing antibody reduced the ability of ASC CM to drive proliferation of SUM159 chemo-residual SUM159 tumor cells by 1.6-fold (Fig. 3C). Likewise this antibody reduced the ability of ASC CM to drive proliferation of chemo-residual BT549 tumor cells by 1.3-fold (Fig. 3D). Collectively, these data show that FGF2 inhibition can suppress the pro-proliferative effects of ASC CM on chemo-residual TNBC cells.

**Fig. 4:**
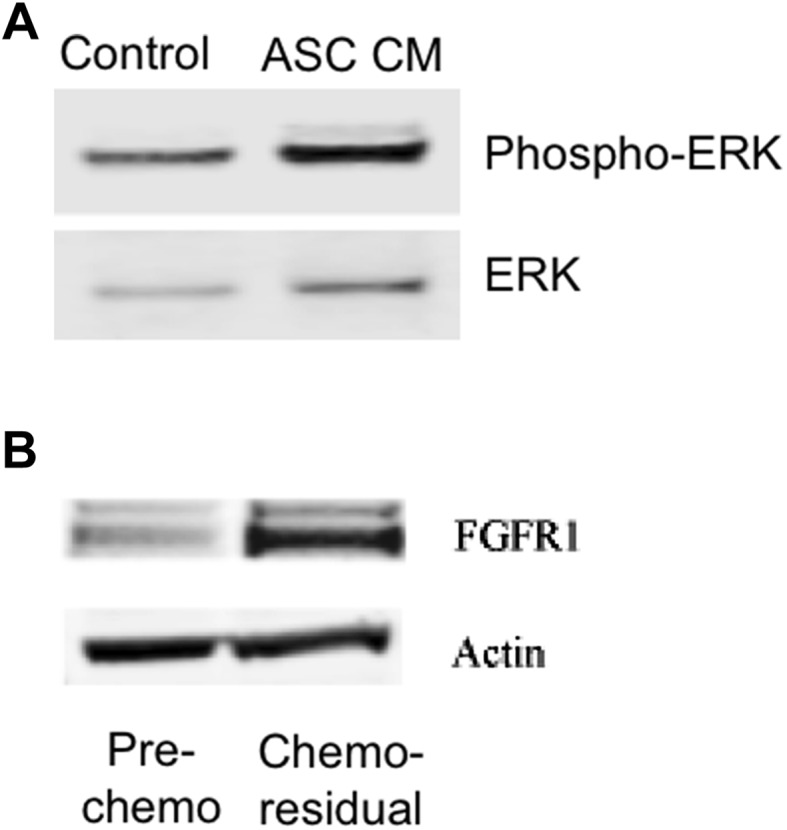
Chemo-residual TNBC signaling. **A** Cytosolic extracts were obtained from chemo-residual SUM159 tumor cells pre-treated ± ASC CM for 24 h. Equivalent amounts were immunoblotted with ERK and phospho-ERK antibodies, followed by the appropriate Alexa Fluor secondary antibody. Protein bands were detected by LI-COR Odyssey Fluorescent imaging. Protein bands were quantified using Image J (NIH) and the ratio of phospho-ERK/ERK for each sample was determined. ASC conditioned media induced a two-fold increase in the phospho-ERK/ERK ratio in chemo-residual cells. **B** Cytosolic extracts were obtained from untreated SUM159 cells and from chemo-residual SUM159 cells. Equivalent amounts were immunoblotted with FGFR1 or Actin antibody, followed by secondary antibody. Protein bands were detected as in **A**

FGF2 drives cell proliferation by activating extracellular signal-regulated kinase (ERK) [15, 16]. Specifically, FGF2 binding to FGF receptors drives tyrosine phosphorylation of ERK, which induces transcription of pro-proliferative and anti-apoptotic proteins [16]. We measured ERK activity in chemo-residual tumor cells following their pre-incubation with ASC CM. ERK activity was measured by determining the ratio of phosphorylated-ERK (phospho-ERK) to ERK in these tumor cells. Using these methods, we demonstrate by western blotting that phospho-ERK: ERK ratios are approximately two-fold higher in chemo-residual tumor cells exposed to ASC CM relative to that in cells exposed to control media (Fig. 4A).

FGF2 signaling is dependent on its binding to one of four FGF2 receptors (FGFR1–FGFR4). Our unpublished data indicate that chemo-residual TNBC cells generated in our short-term chemotherapy treatment model express FGFR1, and that this receptor is important for their survival. Accordingly, we postulated that the differential responsiveness of chemo-residual and chemo-naïve TNBC cells to ASC CM may reflect increased expression of FGFR1 in the chemo-residual cells. To test this hypothesis, we performed FGFR1 immunoblotting on total cellular extracts obtained from SUM159 TNBC cells before and after chemotherapy treatment. As shown in Fig. 4B, chemo-residual SUM159 cells expressed significantly increased levels of FGFR1 relative to chemo-naïve SUM159 cells. Collectively, these data indicate that FGF2 secreted by ASCs acts in a paracrine fashion on FGFR1-expressing chemo-residual TNBC cells to activate ERK signaling, which is associated with an increase in tumor cell proliferation.

## Materials and methods

### Cell culture

SUM159 TNBC cells were obtained from Duke Cell Culture Facility. SUM159 cell line was authenticated (August 2015) with STR profiling at the Duke DNA facility using GenePrint 10 kit (Promega). SUM159 cells were maintained in Ham’s F-12 medium containing 5% heat-inactivated FBS, 5 µg/L insulin, and 1 µg/mL hydrocortisone.

ASCs were purchased from Zenbio (ASC-F), and were maintained in DMEM/F12 medium containing 5% heat-inactivated FBS, penicillin–streptomycin (100 µ/mL), 200 mM l-glutamine and 100 × non-essential amino acids.

BT549 cells were obtained from the Duke Cell Culture Facility and authenticated with STR profiling as above. These cells were maintained in RPMI 1640 containing 10% heat-inactivated FBS, 1 µg/mL insulin, 10 mM HEPES,1 mM pyruvate, and 2.5 g/L glucose.

### Generating chemo-residual cells: short-term chemotherapy treatment

SUM159 or BT549 TNBC cells were cultured for 2 days in Docetaxel (100 nM). After Docetaxel removal, chemo-residual tumor cells were allowed to recover in drug-free complete medium for an additional 16 d. At this time, colonies emanating from chemo-residual tumor cells were harvested with EDTA and expanded as a monolayer for one passage prior to analysis of chemo-residual tumor cell signaling/invasive behavior.

### Transwell chemotaxis assay

Transwell inserts (Costar 3422, 24 well, 8um plate) were coated at 37 °C for 2 h with collagen (Sigma; 25 µg/mL). ASCs were harvested with 0.05% Trypsin–EDTA (Gibco), and washed 3 × with 10 ml FBS free culture medium containing 0.1% BSA. After counting, cells were seeded at 10,000 cells in 100 µl FBS free media + pen/strep + 0.1% BSA into the top chamber of each collagen coated transwell (triplicate wells for each condition). For inhibitor experiments, CXCR4 Ab (10 µg/mL, Invitrogen), AMD3100 peptide inhibitor (50 µg/mL, Millipore Sigma), or control antibody (mouse IgG1, Sigma) were added with cells to top chambers of the transwells.

Conditioned media (CM) from chemo-residual TNBC cells was used as a source of chemo-attractant, and added to the bottom chamber of each transwell. To produce this CM, chemo-residual TNBC cells were grown in FBS free Ham’s/F12 media containing 0.1% BSA, and the supernatant was collected after 48 h. For some experiments (Fig. 2), SDF-1α antibody (10 µg/mL, R&D Systems) or control antibody (mouse IgG1, Sigma) were added to chemo-residual cell CM before plating in the bottom chamber. Plates were incubated at 37 °C/ 5% CO_2_. After 15 h plates were removed and the tops of the transwell inserts were wiped with a Q-tip to remove cells. The inserts were fixed with cold (− 20 °C) Methanol for 10 min and then stained with 0.2 mg /ml Crystal Violet in 2% ethanol for 10 min. Inserts were left to air dry overnight and photographed at 100 ×. The number of migrated cells from 5 fields per insert was counted using cell count in Image J software (NIH). Average number of migrated ASCs from triplicate wells (± SD) was determined for each condition.

### Flow cytometry

ASCs were seeded in a 6 well plate at 1 × 10^5^ cells/well and incubated overnight at 37°C/5% CO_2_. After 24 h, cells were harvested and washed with PBS containing 1% BSA and harvested with a cell scraper. Cells were incubated with CXCR4 antibody (ThermoFisher) or mouse IgG control (Sigma) at a concentration of 1.0 µg/mL at 4 °C for 45 min, followed by secondary antibody (ThermoFisher; 5 µg/mL) at 4 °C for 30 min. Cells were then washed with PBS and analyzed using a Guava flow cytometer and Flowjo software.

### Real-time PCR

RNA was isolated from untreated SUM159 and chemo (docetaxel)-residual SUM159 cell pellets (500,000 cells/pellet) in triplicate using Qiagen RNeasy Mini kit (Cat. No. 74104) following the manufacturers protocol. 500 ng/rxn of isolated RNA was used to make total cDNA with Bio-Rad iScript cDNA synthesis kit (Cat. No. 170-8691) following the manufacturers protocol. SDF-1 Alpha gene expression was determined by RT-PCR using (Accession: NM_199168) Fwd Primer 5′-GTGATTGCCTCTGAAGCC TA-3′ Rev Primer 5′-ATTGTCACCTTGCCAACA GT-3′ and normalized to GAPDH using (Accession: NM_002046.2) Fwd Primer 5′-GAGTCAACGGATTTGGTCGT-3′ Rev Primer 5′-TTGATTTTGGAGGGATCTCG-3′. Gene expression was analyzed by RT-PCR using Bio-Rad SsoAdvanced Universal IT SYBR Green Supermix (Cat. No. 172-5016) according to the manufactures protocol using the Bio-Rad CFX96 Real-Time System and software.

### Proliferation studies

ASC conditioned medium (CM) was produced by culturing ASCs in low serum DMEM/F12 media or control media (DMEM/F12 with 2% FBS) for 72 h. Chemo-residual TNBC cells were seeded into a 96 well plate at a density of 5000 cells/ well. After 24 h, media was removed and chemo-residual TNBC cells were washed with low serum ASC media. Chemo-residual cells were then exposed to ASC CM or control media (DMEM/F12 with 2% FBS). After 24 h, chemo-residual cells wells were harvested with 0.05% Trypsin–EDTA. All experiments were performed in triplicate. Cells were stained with trypan blue and manually counted with a hemocytometer.

In Fig. 4, a fibroblast growth factor 2 (FGF2)-neutralizing antibody (10 µg/mL, EMD Milipore) or isotype control antibody (mouse IgG1, Sigma) were added to the ASC CM before exposure to chemo-residual cells. ASC CM ± FGF2-neutralizing antibody was incubated for 24 h before harvesting and counting cells as above.

### Immunoblotting

Cells were harvested with 2 mM EDTA in HBSS (Gibco) and washed twice with HBSS. Cell pellets were then lysed with Cell Lysis Buffer (CLB) (10 mM Hepes 7.6, 10 mM KCl, 1.5 mM MgCl_2_, 0.5% NP40, PMSF, protease/phosphatase inhibitors (HALT–Pierce). The cytosolic fraction was separated from the nuclear pellet. The nuclear pellet was lysed with Nuclear Lysis Buffer (NLB) (25 mM Tris 7.5, 1% SDS, protease/phosphatase inhibitors). Protein concentrations were determined using Pierce BCA Protein Assay.

Equivalent amounts of cytosolic or nuclear protein were loaded into wells of SDS–PAGE gels (4–12% Bis-Tris Gel, Thermo Fisher), and transferred to nitrocellulose. These membranes were incubated with the following antibodies at 4 °C overnight: Primary antibodies (Cell Signaling: ERK, phosphor-ERK, FGFR1-D8E4. Sigma: Actin). Secondary Antibodies (ThermoFisher: Goat anti Mouse Alexa Fluor 680, Goat anti Rabbit Alexa Fluor 800). Stained membranes were scanned with a LI-COR Odyssey Fluorescent Scanner.

## Discussion

Because TNBC tumors are insensitive to hormonal therapy, chemotherapy is the standard of care for women diagnosed with these aggressive cancers. Our laboratory has demonstrated that triple-negative breast cancers are heterogeneous, composed of both chemo-sensitive and chemo-resistant subpopulations of tumor cells [5]. Following chemotherapy treatment and the death of chemo-sensitive cells, a population of chemo-residual cells remain viable. Our previous work demonstrates that chemo-residual cells have a slower proliferation rate than the bulk tumor population [6]. The reduced proliferation of these cells in part drives their resistance to chemotherapeutic agents, which rely on rapid cell proliferation for efficacy. Restored proliferation of this dormant population by unknown factors occurs years after chemotherapy treatment, driving recurrent tumor growth.

**Fig. 5:**
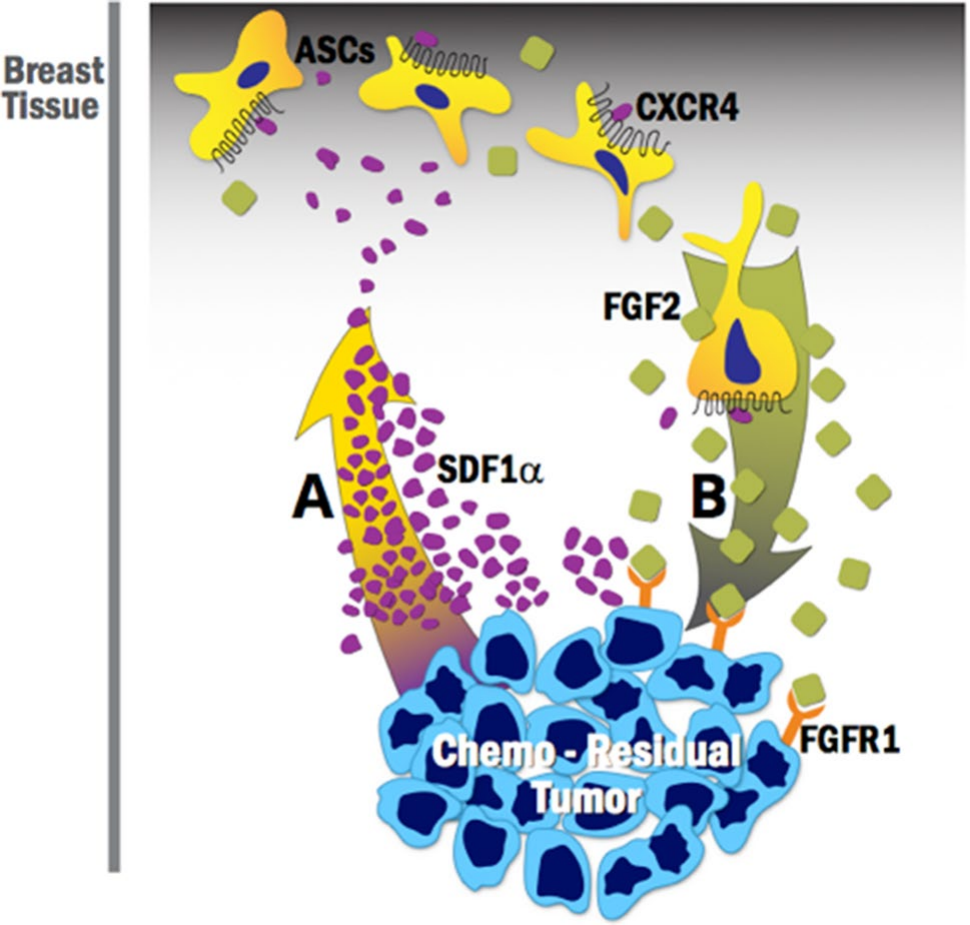
ASC cross-talk with chemo-residual TNBC cells- implications for tumor recurrence. **A** Chemo-residual TNBC cells secrete the chemokine SDF1α, resulting in recruitment of ASCs, which express the SDF1α chemokine receptor CXCR4, to the tumor site. **B** ASCs secrete FGF2, which binds to an FGF2 receptor on chemo-residual tumor cells, initiating signaling that drives tumor cell proliferation. Considering that chemo-residual tumor cells can remain dormant in patients for months to years, this ASC/chemo-residual tumor cell cross-talk likely contributes to tumor recurrence in patients post-chemotherapy treatment

Our study demonstrates that chemo-residual cells secrete a chemokine (SDF1α) that binds to ASCs in the breast stroma, and recruits them to the chemo-residual tumor cell microenvironment via the SDF1α-CXCR4 chemotactic axis (Fig. 5A). Our results show that blocking this axis only partially reduces ASC migration (Fig. 2), suggesting that other chemotactic axes are operative. Once within the tumor microenvironment, these ASCs secrete a factor (FGF2) that acts in a paracrine fashion on chemo-residual TNBC cells to induce their proliferation (Fig. 5B). Our work shows that ASC-driven proliferation of chemo-residual tumor cells is dependent on their secretion of FGF2. However, even in the presence of the FGF2-neutralizing antibody, proliferation rates did not completely return to baseline levels (Fig. 3). This finding suggests that ASCs may release additional growth factors and cytokines into the tumor microenvironment that together with FGF2, promote tumor cell proliferation. Identifying these additional growth factors is a subject of future investigation.

Approximately 60% of TNBC patients exhibit an incomplete pathologic response to neoadjuvant chemotherapy, as indicated by the presence of residual chemo-resistant cancer cells [3, 17]. The presence of chemo-residual TNBC cells is associated with a high rate of tumor recurrence, which is responsible for patient mortality [4]. Chemo-residual tumor cells can remain in a dormant state for many years prior to the growth of locoregional or distant recurrent tumors. It remains unclear what factors transition these tumor cells from dormancy to recurrence. Notably, chemo-residual tumor cells are exposed to an adipose rich environment within the breast both before surgery and after breast reconstruction. Our work demonstrates that the transition from dormancy to recurrence may be influenced by ASC secretion of growth factors acting in a paracrine fashion to drive chemo-residual TNBC cell proliferation. To address this possibility, in future studies, we will test if co-injection of ASCs with chemo-residual tumor cells into immunodeficient mice accelerates tumor growth kinetics.

FGF2 induces cell proliferation by binding to FGF receptor family members. Our unpublished data indicate that chemo-residual TNBC cells generated in our short-term chemotherapy treatment model express FGFR1, and that this receptor is important for their survival. In the current work, we show that chemo-residual TNBC cells express increased levels of FGFR1 compared to chemo-naïve cells, providing mechanistic insight into why chemo-residual but not chemo-naïve TNBC cells proliferate in response to their incubation with FGF2-secreting ASCs. Further studies are needed to determine if small molecule FGFR inhibitors used in the clinic can inhibit ASC/chemo-residual TNBC cell cross-talk.

Previous studies indicate that activation of FGF2/FGFR induces proliferation and chemo-resistance of solid tumors [15, 18]. Upon receptor activation, downstream signaling promotes the phosphorylation of extracellular signal-regulated kinase (ERK), which drives cell growth and proliferation in many tumor types via the upregulation of pro-proliferative and anti-apoptotic proteins [16]. In the present study, we demonstrate by immunoblotting that chemo-residual TNBC cells exposed to ASC CM express increased levels of phospho-ERK. These data provide mechanistic evidence for the anti-proliferative effects observed when FGF2 signaling is impaired. In addition to its pro-proliferative effects, FGF2 can also promote chemotherapy resistance by activating ERK [19]. Future studies are needed to: (1) investigate if ASC-driven paracrine FGF2 signaling contributes to the development of TNBC cell chemo-resistance, and (2) determine if FGFR inhibitors can restore chemo-sensitivity of these TNBC cells.

FGF2-neutralizing antibodies block FGF2 binding to FGF receptors. In the present study, we showed that an FGF2- neutralizing antibody blocks ASC CM-induced chemo-residual TNBC cell proliferation (Fig. 3). Other strategies for inhibiting FGF2 signaling include the use of a fusion protein, FGF-Trap, which acts as a soluble decoy FGF2 receptor. FGF-Trap has proven effective in the inhibition of growth and angiogenesis of tumors in vivo [20]. An additional method for blocking this signaling axis includes the use of a tyrosine kinase inhibitor, NVP-BGJ398, which inhibits the activation of FGFRs [21] and impairs breast cancer progression [22]. Finally, this signaling can be inhibited using B19, a myristoyl-CoA analog that prevents the myristoylation of fibroblast growth factor receptors substrate 2 (FRS2α), a key scaffolding protein necessary for receptor functionality. Recent data suggest B19 interferes with downstream FGF2 signaling via alterations to MAPK, triggering cell cycle arrest and halting tumor cell proliferation [23].

FGFR tyrosine kinase inhibitors are used as a treatment modality in patients to slow the growth of tumors across a wide array of cancer types including breast, prostate, lymphoma, multiple myeloma, and urothelial carcinoma [24]. The classes of compounds presently in use or in clinical trials are selective and non-selective tyrosine kinase inhibitors, such as BGJ398, as well as monoclonal antibodies and FGF-ligand traps described previously. To qualify for treatment or to participate in clinical trials with these agents, patients must meet eligibility criteria, including possession of known FGFR mutations, amplifications, or translocations [24]. In the present study, we demonstrate that FGF2 signaling from stromal cells plays a key role in supporting the tumor microenvironment suggesting that many patients that do not presently meet eligibility criteria for FGFR inhibitor treatment may in fact benefit from these drugs because of their ability to inhibit ASC paracrine signaling.

In summary, this study suggests that the breast stroma can influence chemo-residual TNBC cells remaining after neoadjuvant chemotherapy treatment, thus potentially contributing to tumor recurrence. Our work suggests that disrupting the supportive role that ASCs provide for tumor cells, either by preventing ASC migration to the chemo-residual tumor microenvironment (Fig. 5A) or by inhibiting the signaling associated with ASC release of growth factors such as FGF2 (Fig. 5B), may prevent TNBC recurrence. Given the resistance of these cells to chemotherapy, furthering our understanding of ASC/chemo-residual tumor cell cross-talk, and its role in recurrent tumor formation will provide an essential foundation for developing more effective therapeutic approaches.
